# The apparent diffusion coefficient as a potential radiological biomarker of malignant transformation in retinoblastoma: a case report

**DOI:** 10.1007/s00247-025-06451-8

**Published:** 2025-11-18

**Authors:** Sonia De Francesco, Paolo Galluzzi, Tommaso Padula, Giovanni Rubegni, Pierluigi De Marzo, Mario Fruschelli, Doris Hadjistilianou

**Affiliations:** 1 Ophthalmology Unit, Department of Medicine, Surgery and Neurosciences, University of Siena, Viale Bracci 1, 53100 Siena, Italy; 2 Department of Medicine, Surgery and Neuroscience, Unit of Ophthalmology Ocular Oncology – Retinoblastoma Referral Center, Azienda Ospedaliera Universitaria Senese, Viale Bracci 1, 53100 Siena, Italy; 3 Department of Neuroimaging and Neurointervention, Siena University Hospital, Viale Bracci 1, 53100 Siena, Italy

**Keywords:** Amblyopia, Esotropia, Infant, Magnetic resonance imaging, Ophthalmoscopy, Retinoblastoma

## Abstract

We report a unique case of malignant transformation in a retinocytoma monitored using magnetic resonance imaging (MRI). This case is notable for a distinct drop in apparent diffusion coefficient (ADC) values that correlated with clinical transformation to retinoblastoma. This is the first report to highlight a change in ADC as a radiological marker of malignant conversion in retinocytoma. This finding suggests the potential role of ADC in prognostic assessment and risk stratification for patients with retinocytoma.

## Introduction

Retinocytoma is a rare, benign retinal tumor often considered a spontaneously regressed form of retinoblastoma [1]. Several case reports have described its clinical and ophthalmoscopic features, including calcified vitreous deposits [2]. While typically stable, retinocytoma may undergo malignant transformation above all in the first three years of life [3]. Differentiating between retinocytoma and retinoblastoma remains a challenge, particularly when early transformation lacks overt clinical features. Imaging, especially magnetic resonance imaging (MRI) [4] with diffusion-weighted imaging (DWI), is promising in evaluating these lesions.

## Case report

**Fig. 1:**
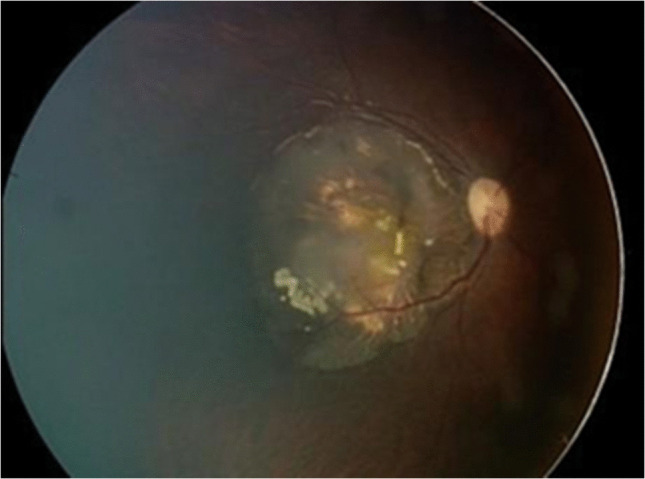
Fundus image of a 19-month-old girl, previously diagnosed with retinocytoma. A partially calcified cystic lesion with a translucent component is observed covering the posterior pole

A 19-month-old female presented with right eye esotropia and amblyopia. Ophthalmoscopy revealed a translucent, calcified mass (6.5×2.3×5 mm) at the posterior pole with two cystic areas and no feeder vessels (Fig. 1). The lesion was consistent with retinocytoma. Genetic analysis identified a P.T307I retinoblastoma mutation.

**Fig. 2:**
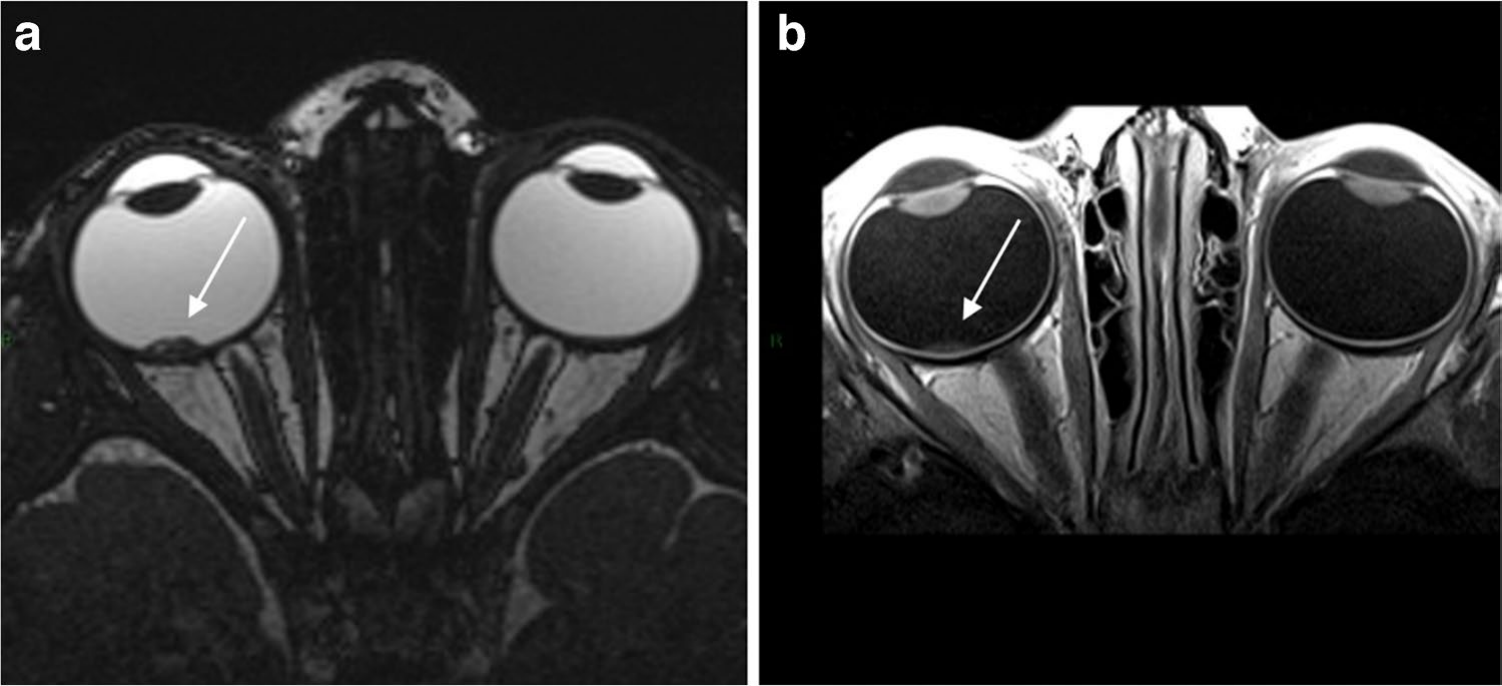
MRI of a 19-month-old girl at the time of imaging, diagnosed with retinocytoma. Axial T2-weighted (**a**) and post-contrast T1-weighted (**b**) images show (*arrow*) a slightly enhancing macular mass. *MRI*, magnetic resonance imaging

MRI was performed at diagnosis (Fig. 2), 6 months later, when malignant transformation was suspected on ophthalmoscopy and during post-remission follow-up.

Axial T1- and T2-weighted high-resolution images were obtained. Additionally, diffusion-weighted images (b 0–1000) with apparent diffusion coefficient map and T1-weighted axial high-resolution images after paramagnetic contrast medium intravenous injection were performed.

Initial MRI demonstrated elevated ADC values (>1×10^−3^ mm^2^/s), higher than typical retinoblastoma thresholds [5].

After 11 months of follow-up, the lesion remained stable. The situation was assessed monthly by ophthalmoscopy and every 6 months by MRI, too.

**Fig. 3:**
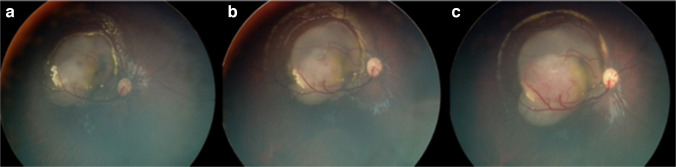
Fundus images showing malignant transformation of retinocytoma over time. The progression starts 11 months after diagnosis (age 30 months) and continues over the following 2 months: at 30 months (**a**), 31 months (**b**), and 32 months (**c**)

At 30 months, a small dome-shaped nodular outgrowth and a dilated feeder vessel appeared, indicating malignant transformation (Fig. 3).

**Fig. 4:**
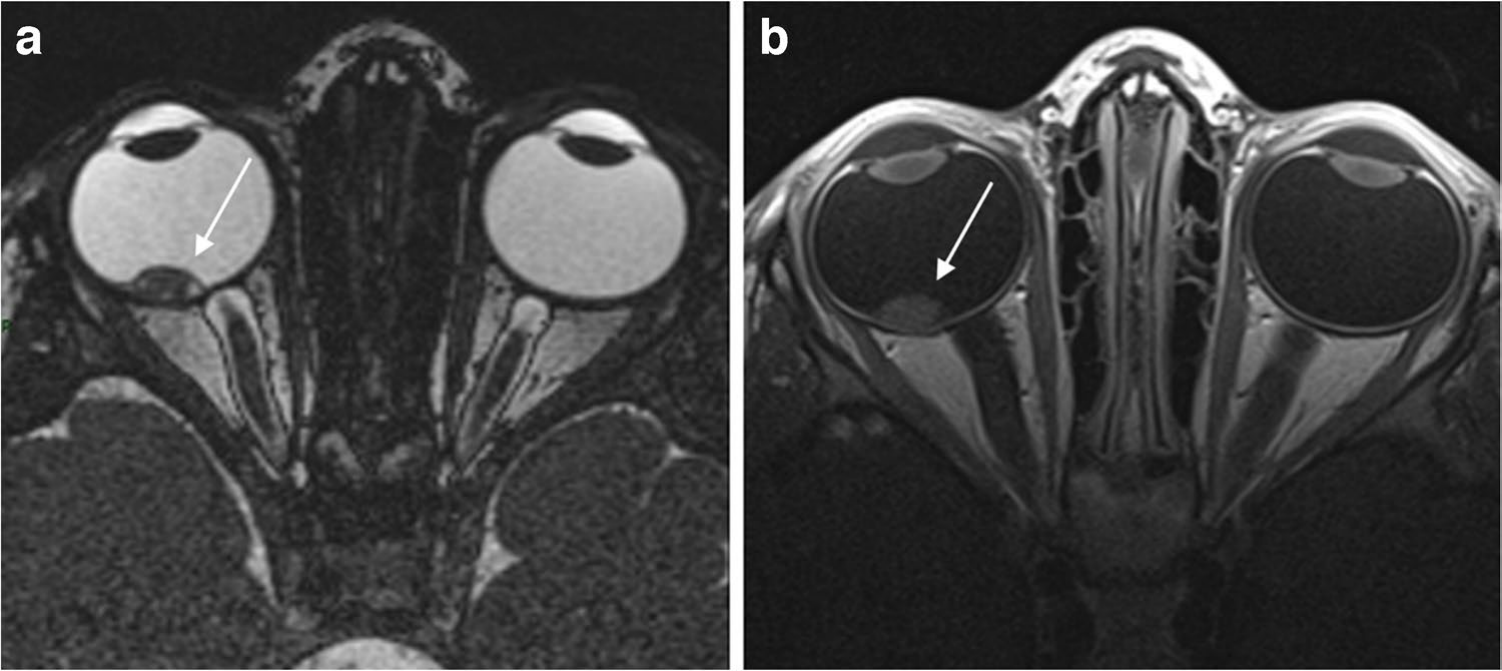
MRI of a 30-month-old girl at the time of imaging, previously diagnosed with retinocytoma. Axial T2-weighted (**a**) and post-contrast T1-weighted (**b**) images show (*arrow*) an increase in size and degree of contrast enhancement of the macular mass compared with prior imaging. *MRI*, magnetic resonance imaging

**Fig. 5:**
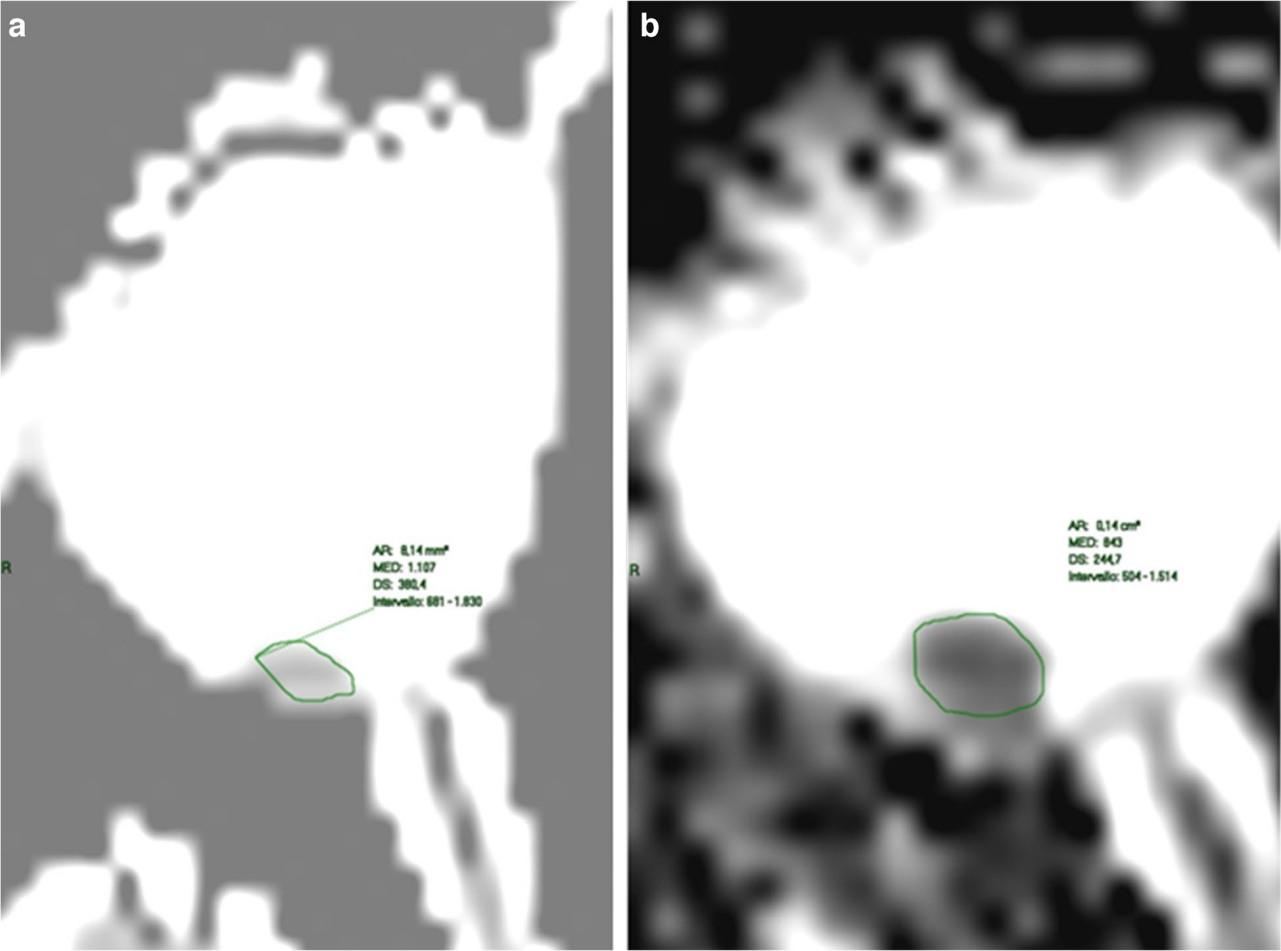
Axial diffusion-weighted MRI of a girl at 19 months (**a**) and 30 months (**b**), previously diagnosed with retinocytoma. The first scan (**a**) demonstrates a high ADC value, whereas the subsequent scan (**b**) shows a reduced ADC value, reflecting changes in the tumor over time. *MRI*, magnetic resonance imaging

Subsequent MRI revealed a drop in ADC (0.85×10^−3^ mm^2^/s), consistent with increased tumor cellularity and an increase in lesion size (Figs. 4 and 5).

The patient received four cycles of intra-arterial chemotherapy (melphalan and topotecan). The malignant portion regressed, while the original retinocytoma component remained unchanged.

**Fig. 6:**
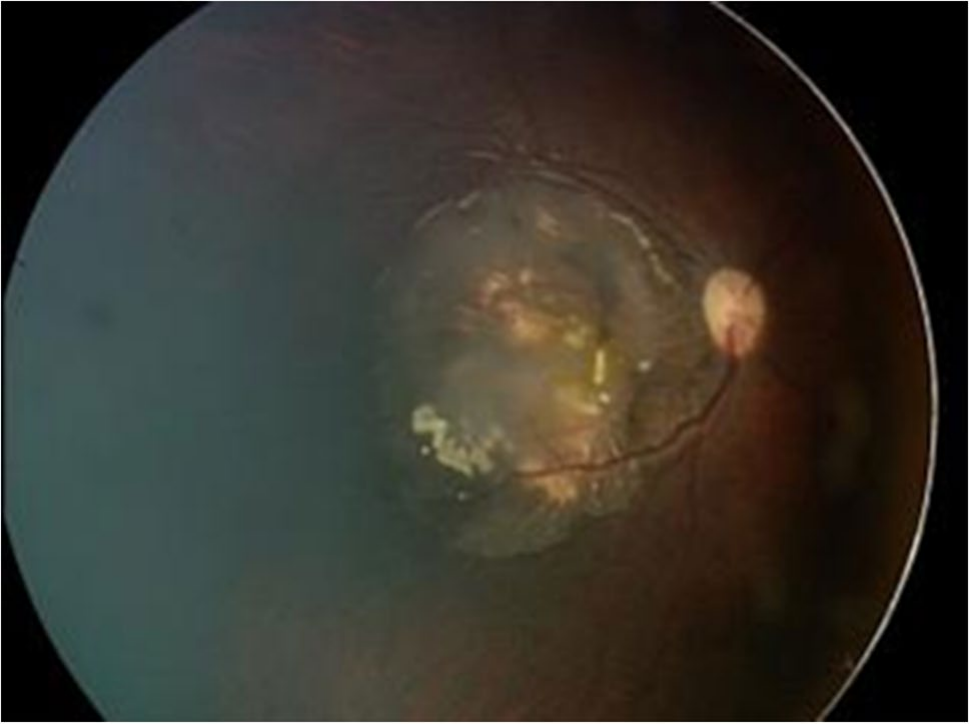
Fundus image of a 6-year-old girl with amblyopia and esotropia. Four months after intra-arterial chemotherapy with melphalan and topotecan, the transformed lesion has regressed, while the main bulk of the tumor remains unchanged (regression pattern type III)

At 4-year follow-up, the lesion was stable with no evidence of recurrence (Fig. 6).

## Discussion

This case highlights the utility of ADC in detecting malignant transformation [3] of retinocytoma. ADC reflects water diffusion and tissue cellularity; lower values are associated with highly cellular, undifferentiated tumors like retinoblastoma. In this patient, a clear decline in ADC values [5] corresponded with clinical transformation, preceding histologic confirmation.

According to our protocol, we performed an MRI at the time of diagnosis, 6 months after diagnosis and when malignant transformation was detected by ophthalmoscopy. In our case, the ADC value decreased markedly at the time of ophthalmoscopic transformation and returned to the baseline value following treatment, mirroring the changes observed in the ophthalmoscopic appearance. The active component of the lesion regressed, and the lesion recovered its initial, inactive appearance. According to this reasoning, if a decrease in the ADC value is observed during follow-up, it may be suspected that the lesion is transforming into retinoblastoma. Therefore, based on the ADC values, it may be decided to bring forward follow-up visits or even initiate preventive therapy, even in the absence of ophthalmoscopically detectable changes.

While ophthalmoscopy remains essential, MRI [4] and specifically ADC mapping may provide noninvasive insights into tumor behavior. Post-treatment changes in ADC [6] further support its value as a dynamic biomarker in retinoblastoma management. The constancy of ADC in the remaining retinocytoma suggests specificity for malignant areas.

This is the first report correlating ADC reduction with retinocytoma transformation, supporting its use as a potential radiologic biomarker for malignancy risk. Measuring ADC with magnetic resonance imaging does not eliminate any other methods, but it may be an indicator of malignant transformation.

This report is limited by its single-case nature and the fact that the decrease in ADC was observed only after clinical signs of malignant transformation. ADC values lack an absolute cutoff and may vary with necrosis and MRI field strength, so our findings should be interpreted with caution. A reduction in ADC may suggest, but not definitively prove, malignant progression and must always be correlated with clinical findings.

## Data Availability

No datasets were generated or analysed during the current study.
